# Integrating Non-human Primate, Human, and Mathematical Studies to Determine the Influence of BCG Timing on H56 Vaccine Outcomes

**DOI:** 10.3389/fmicb.2018.01734

**Published:** 2018-08-17

**Authors:** Louis R. Joslyn, Elsje Pienaar, Robert M. DiFazio, Sara Suliman, Benjamin M. Kagina, JoAnne L. Flynn, Thomas J. Scriba, Jennifer J. Linderman, Denise E. Kirschner

**Affiliations:** ^1^Department of Chemical Engineering, University of Michigan, Ann Arbor, MI, United States; ^2^Department of Microbiology and Immunology, University of Michigan Medical School, Ann Arbor, MI, United States; ^3^Department of Microbiology and Molecular Genetics, University of Pittsburgh School of Medicine, Pittsburgh, PA, United States; ^4^South African Tuberculosis Vaccine Initiative and Institute of Infectious Disease and Molecular Medicine, Division of Immunology, Department of Pathology, University of Cape Town, Cape Town, South Africa

**Keywords:** tuberculosis, non-human primate, H56, mathematical modeling, bacillus calmette–guerin (BCG), vaccination

## Abstract

Tuberculosis (TB) is the leading cause of death by an infectious agent, and developing an effective vaccine is an important component of the WHO's EndTB Strategy. Non-human primate (NHP) models of vaccination are crucial to TB vaccine development and have informed design of subsequent human trials. However, challenges emerge when translating results from animal models to human applications, and connecting post-vaccination immunological measurements to infection outcomes. The H56:IC31 vaccine is a candidate currently in phase I/IIa trials. H56 is a subunit vaccine that is comprised of 3 mycobacterial antigens: ESAT6, Ag85B, and Rv2660, formulated in IC31 adjuvant. H56, as a boost to Bacillus Calmette-Guérin (BCG, the TB vaccine that is currently used in most countries world-wide) demonstrates improved protection (compared to BCG alone) in mouse and NHP models of TB, and the first human study of H56 reported strong antigen-specific T cell responses to the vaccine. We integrated NHP and human data with mathematical modeling approaches to improve our understanding of NHP and human response to vaccine. We use a mathematical model to describe T-cell priming, proliferation, and differentiation in lymph nodes and blood, and calibrate the model to NHP and human blood data. Using the model, we demonstrate the impact of BCG timing on H56 vaccination response and reveal a general immunogenic response to H56 following BCG prime. Further, we use uncertainty and sensitivity analyses to isolate mechanisms driving differences in vaccination response observed between NHP and human datasets. This study highlights the power of a systems biology approach: integration of multiple modalities to better understand a complex biological system.

## Introduction

Among infectious diseases, tuberculosis (TB) remains the leading cause of death due to a single agent. Its infectious agent, *Mycobacterium tuberculosis* (Mtb), kills approximately three individuals per minute (WHO, 2016). Additionally, in 2015, there were an estimated 480,000 incident cases of multi-drug resistant TB. The morbidity and mortality due to tuberculosis, including drug resistant strains, require renewed investment and research for an effective vaccine.

While Bacillus Calmette-Guérin (BCG) is widely used to prevent TB disease in infants, its efficacy amongst the adult population is highly variable (Colditz et al., 1995; Fine, 1995; Lanckriet et al., 1995; Mittal et al., 1996; Sterne et al., 1998; Zodpey et al., 1998). Originally developed in the early 1900s, the first clinical trials for BCG began in France in the 1920s and proved its efficacy in children (Andersen and Doherty, 2005). By 1973, BCG was compulsory for South Africa (Fourie, 1987) and emerged as the most widely used of all vaccines, due to ease of testing for vaccination via the tuberculin skin test. However, BCG efficacy fails to protect both infants and adults; with protection varying from 0-80% (Andersen and Doherty, 2005; Tameris et al., 2013). Thus, the search for a more effective vaccine continues.

Improved management of the TB epidemic could stem from vaccinations that prevent infection, active disease, or reactivation from latent infection, or ameliorate active infections. Currently, more than 13 TB vaccine candidates have entered clinical trials (Evans et al., 2016; Gonzalo-Asensio et al., 2017). These candidates include attenuated versions of Mtb, mycobacterial whole cell vaccines, viral vectored vaccines, and subunit vaccines (Ahsan, 2015).

Subunit vaccination strategies emerged when the Mtb genome was sequenced in 1998 (Cole et al., 1998). One such promising subunit vaccine candidate is H56 formulated with adjuvant IC31. H56 is a multistage vaccine composed of three antigens: ESAT6, Ag85B, and Rv2660c (Aagaard et al., 2011). ESAT6 and Ag85B are early secreted antigens that have been used before as individual vaccine antigens (Horwitz et al., 1995; Brandt et al., 2000; Olsen et al., 2001, 2004; Langermans et al., 2005). Ag85B is an antigen that is present in both BCG and H56 vaccine formulations. Both Ag85B and ESAT6 have been shown to be highly immunogenic antigens that are targeted by T cell populations (Mustafa et al., 2000a,b). Rv2660c was included in the vaccine because of its association with T cell responses from LTBI (Latent Tuberculosis Infection) individuals and its expression under starvation or hypoxic conditions, although its function has not yet been determined (Betts et al., 2002; Govender et al., 2010; Lin et al., 2012). Finally, all three antigens are thought to play a role in a variety of methods that mycobacteria likely employs to survive the intracellular environment (Ronning et al., 2000; Wilkinson et al., 2001; Ganguly et al., 2008; Lin et al., 2012; Rohde et al., 2012).

Common formulations of the H56 vaccine include the adjuvants IC31 and Cationic Adjuvant Formulation (CAF01). Human clinical trials used the IC31 adjuvant, a two-component adjuvant that includes the KLK peptide (an anti-microbial peptide) and oligodeoxynocleotide (a Toll-like receptor nine agonist) (Luabeya et al., 2015). IC31 was used in an NHP study that showed H56 limited reactivation of clinical latent TB (Lin et al., 2012), while CAF01 has been used in NHP studies herein. CAF01 is composed primarily of DDA (liposomes prepared in dimethyl dioctadecyl ammonium) and TDB (a component of the mycobacterial cell wall, trehalose dimycolate) (Agger, 2016). Both adjuvants support a Th1 CD4 T cell response (Luabeya et al., 2015; Agger, 2016).

While H56 represents a new vaccine candidate, it also provides an opportunity for a case study. Before evaluating the success of a vaccine via challenge, can we compare vaccine immunogenicity in humans and NHPs to further characterize the inherent differences between each species? Furthermore, can we utilize antigen specificity to explore the impact and role of prior BCG vaccination on H56 immunogenicity?

We use a systems biology approach employing mathematical modeling to relate pre-exposure vaccination dynamics in humans and non-human primates. We describe T-cell responses in lymph nodes and blood using a 2-compartment mathematical model, demonstrate the impact of BCG timing on subsequent H56 vaccination, and reveal basic mechanisms that dictate vaccine outcomes in NHPs and humans. We propose that timing of BCG vaccination and inherent differences between species could play an important role in the immune responses to the H56 vaccine candidate. Having this knowledge could improve the vaccine pipeline.

## Methods

### Non-human primate data collection and analysis

#### Animals

The Institutional Animal Care and Use Committee of the University of Pittsburgh approved all experiments (protocol number 12080653). The animals were housed and maintained in accordance with standards established in the Animal Welfare Act and the Guide for the Care and Use of Laboratory Animals.

#### Vaccination

Cynomolgus macaques (*Macaca fascicularis*) imported from China and in the United States for at least a year (Valley Biosystems) were used for these studies (n = 8). BCG and H56:CAF01 animals were primed with 0.1 mL BCG Danish intramuscularly followed by two doses of the vaccine H56 (Ag85B-ESAT6-Rv2660c; 50 μg) mixed with CAF01 (625 μg dimethyldioctadecyl-ammonium (DDA) and 125 μg trehalose-6,6-dibehenate (TDB)) at weeks 10 and 14 after BCG priming. Timing and doses of vaccination are based on previous studies by our collaborators and others in the field who perform protein-based boosting of BCG in macaques (Langermans et al., 2005; Lin et al., 2012).

#### Necropsy

For this study, macaques were euthanized approximately 44-48 weeks post-BCG prime (macaques received Mtb challenge 22 weeks following BCG prime, but Mtb-challenge data response was not included in this study and is therefore not outlined in this section). All animals were euthanized with an intravenous overdose of sodium pentobarbital (Beuthanasia) at 15mg/kg and maximally bled.

#### ELISPOT

ELISPOT for IFN-γ was performed using 96-well opaque multiscreen immunoprecipitation filtration plates (Merck Millipore) that were hydrated, washed, and coated with 7.5 μg/mL of anti-human/nonhuman primate IFN-γ (GZ-4: Mabtech) for 2 h at 37°C with 5% CO_2_. Plates were then blocked with complete RPMI containing 10% human AB serum for 2 h at 37°C with 5% CO_2_. Each stimulation condition was performed in duplicate. Medium only was used as a negative control, and phorbol dibutyrate/ionomycin (P&I) and anti-CD3 were used as positive controls. CFP and peptide pools of H56 vaccine antigens (ESAT-6, Ag85B, Rv2660c) were used at 10 μg/mL. PBMCs were then added, and the plate was incubated for 48 h at 37°C with 5% CO_2_. The plate was then washed and detection antibody (7-B6: Mabtech) was added at 2.5 μg/mL and incubated for 2 h at 37°C with 5% CO_2_. The plate was washed and streptavidin-conjugated horseradish peroxidase was added at a 1:100 dilution and incubated for 45 min at 37°C with 5% CO_2_. The plate was washed and then developed using AEC substrate. The plate was dried overnight and read using an ImmunoSpot analyzer (Cellular Technologies Limited).

**Figure 1:**
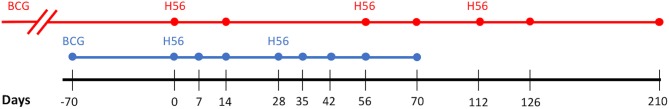
Vaccination Experimental Protocol. Comparison of the Human (red) and Non-Human Primate (blue) study protocols. Dots along the respective timelines represent blood sample data collection time points. BCG, Bacillus Calmette–Guerin; H56, vaccination with H56 and adjuvant (IC31 in Human, CAF01 in Non-Human Primate).

Figure 1 shows the timeline of experimental protocol, with blood draw events for NHP studies (bottom timeline). We represent the data from Difazio et al. in a manner consistent with the standardization of the phase I clinical trial data provided by Luabeya et al. Like Luabeya et al., we analyzed the antigen specific T cell response for CD4+ effector (CD27-CD45+), effector memory (CD27-CD45-), and central memory (CD27+CD45-) subtypes. ESAT6- or Ag85B-specific cellular concentrations were calculated. Finally, we converted the antigen-specific responses for each T-cell subtype to represent a percentage of total CD4+ T cells in blood.

### Phase I clinical trial data collection and analysis

For model calibration, we used data described previously (Luabeya et al., 2015). Briefly, the data is from the first in-human phase I clinical trial of candidate TB vaccine, H56 in IC31 adjuvant. The authors tested the safety and immunogenicity of H56:IC31 in adults with or without Mtb infection. Across 112 days, eight individuals without evidence of Mtb infection were injected with 3 doses of H56 (50 μg H56, 500 nmol IC31) at 56 day intervals. Blood was drawn from individuals on days 0, 14, 56, 70, 112, 126, and 210. Antigen-specific T-cell responses were isolated and collected at each sample collection time point. Every individual in the study received BCG vaccination as a child (approximately 30 years prior to this study). Figure 1 shows the timeline of experimental protocol for the human trial (top timeline).

We standardized the results of Luabeya et al. in a manner that allows for eventual comparison to NHP data. The study revealed that the H56 vaccine does not induce a robust CD8+ T cell response. Therefore, we focused all data analysis, model calibration, and results on individual subtypes of the CD4+ T cell response to vaccination. That is, we examined effector (CD45RA^+^ CCR7^−^), effector memory (CD45RA^−^CCR7^−^), central memory (CD45RA^−^CCR7^+^), and total CD4+ T cell populations. Luabeya et al. also discovered that a dose of 50 μg of H56 was not optimal; however, we have selected the 50 μg dataset so that we can directly compare human responses to the NHP studies described above.

For each T cell subtype, we normalized the response by subtracting the number of unstimulated, cytokine-producing T cells from the quantity of T cells that produced cytokines in response to antigen. We converted this metric to represent a percentage of the total number of CD4+ T cells. This calculation was performed for responses to both the ESAT6 and Ag85B antigens.

Note that the adjuvants used in these two studies (NHP and human) are different and could contribute significantly to the results observed. In this work, we do not examine adjuvant differences but focus instead on the impact of BCG timing and differences in T cell responses between species. See below for further discussion of how we indirectly capture adjuvants.

### Mathematical model

**Figure 2:**
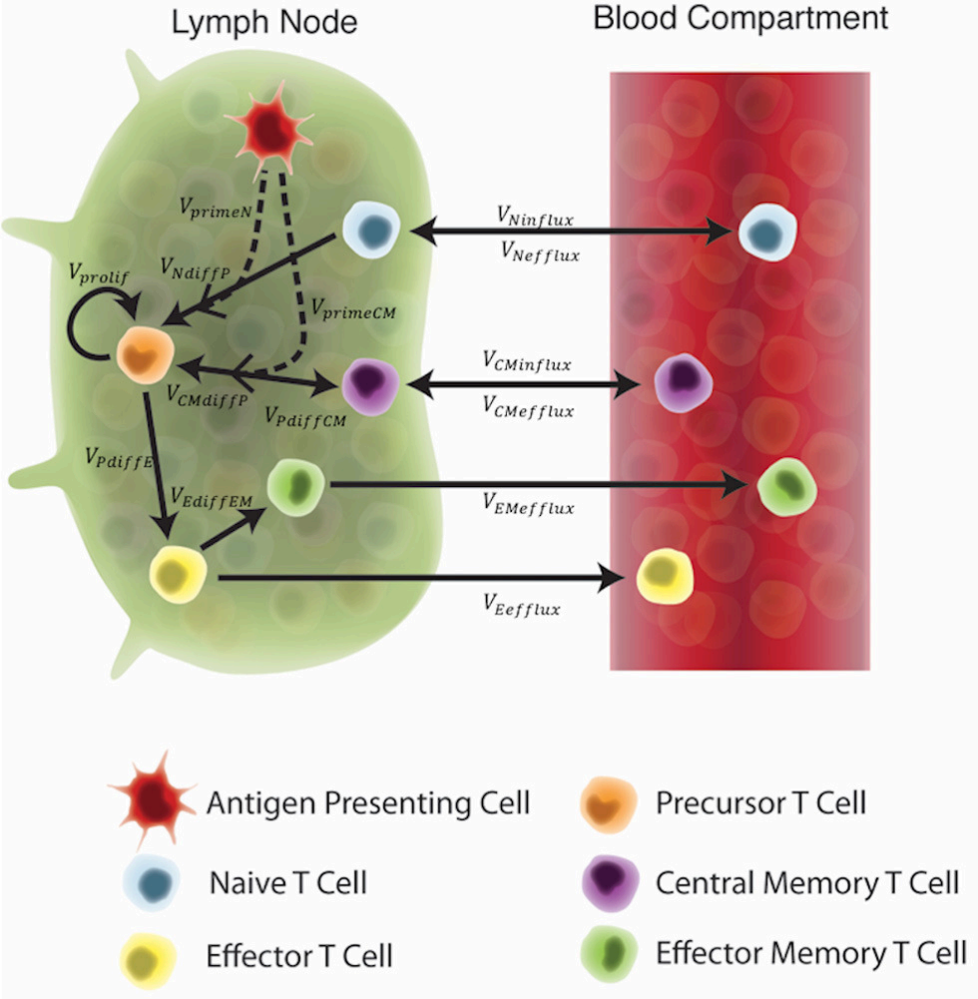
Schematic of the two-compartment model. Each equation represents a concentration of a particular cell type, as outlined in the legend. These concentrations are dependent on other cell concentrations and interactions (as shown by arrows) between cells or compartments. Arrow labels are defined in greater detail in Supplementary Materials 1. Briefly, *V*_*primeN*_ and *V*_*primeCM*_ represents the impact of APCs on naïve and central memory cell recruitment. *V*_*NdiffP*_ and *V*_*CMdiffP*_ shows the transformation of naïve and central memory T cells to the precursor T cell population. *V*_*prolif*_, *V*_*PdiffE*_, *V*_*PdiffCM*_, and *V*_*EdiffEM*_ represents precursor proliferation and differentiation to effector, central memory and effector memory cell types, respectively. Finally, influx and efflux rates between LN and blood are shown as *V*_*Ninflux*_, *V*_*CMinflux*_, *V*_*CMefflux*_, *V*_*Eefflux*_, and *V*_*EMefflux*_.

In recent studies (Gong et al., 2014; Marino and Kirschner, 2016; Marino et al., 2016; Ziraldo et al., 2016), we captured lymph node and blood dynamics in response to Mtb infection using a mathematical model. We used a compartmentalized system of 16 non-linear, autonomous ordinary differential equations (ODEs) to track specific and non-specific CD4+ effector, effector memory, and central memory T cell responses. In these previous works we represent Mtb-specific T-cells as a generic class of antigen-specific cells; thus, it was simple to retool this class of cells and track them as ESAT6- or Ag85B-specific. We assume that all antigen-specific T cells are equally immune responsive. Figure 2 displays the model schematic, Supplementary Materials 1 details the system of ODEs, and Supplementary Table 1 gives the list of all parameters, definitions, and values.

Our key assumption is that the *in silico*, exogenous introduction of antigen loaded, antigen-presenting cells (APCs) will act as a reasonable proxy for vaccination. This is valid for two reasons: First, it is well known that vaccine peptides are presented to T cells by APCs. Second, while we did not mechanistically model the impact of an adjuvant in this study, this assumption indirectly evaluates the impact of an adjuvant on T-cell responses. APCs require adjuvant to properly process and present vaccine peptides (Kamath et al., 2008). Therefore, to account for variability in individual response to an adjuvant and to represent variability across adjuvants (IC31 vs. CAF01), the quantity of APCs pulsed during vaccination events was assigned to a single quantity within a range of values. Thus, we simulated vaccination events by pulsing the APC equation in the system of ODEs at a time point equal to the day of H56 vaccination, according to each experimental protocol.

The non-linear ODE model system was implemented and solved in Matlab (R2016b v 9.1). Experimental and simulation data cleaning, visualization, and post-processing was performed in R (R version 3.4.0, RStudio version 1.0.143) using ggplot2 (Wickham, 2009), plyr (Wickham, 2011), and tidyr (Wickham and Henry, 2017) packages. See Supplementary Materials 1 for equations and model parameters.

### Model calibration and sensitivity analysis

We first sought to define the parameter space that best represents each “immunogenicity dataspace” to calibrate to the human and NHP datasets (see Box 1 for a description of several important terms for this section of our work). The parameter space was identified by a two-step process. First, for each immunogenicity space, we ran 1500 simulations with a 50% range around the baseline parameters outlined in our previous model construction (shown in Marino and Kirschner, 2016). A Latin hypercube sampling (LHS) algorithm was used to sample the multi-dimensional parameter space (Marino et al., 2008). This wide range of simulations yielded multiple candidates of baseline parameters that might best represent each immunogenicity dataspace. In the second step, we simulated 500 runs (sampling parameters in approximately 20% range) around these candidates' baseline values, again using LHS to sample the parameter space. We accepted the candidate parameter sets if all 500 runs fulfilled two criteria: (1) the simulations' minimum and maximum run must remain within the immunogenicity dataspace. That is, all simulations from the parameter ranges needed to remain within the logarithmic scale of the data. (2) the median simulation run across all 500 runs must cross the interquartile range of the majority of experimental time points (4 of 7 for human data, 4 of 8 for NHP data). This ensured that our model mimics at least the majority of both experimentally-determined dynamics. Supplementary Table 1 displays the parameter range values after calibration to each immunogenicity dataspace.

Box 1 **Important terms**

**Immunogenicity Dataspace:** The space defined by experimental results that contains the T-cell response to each antigen.

**Parameter Range:** The range of values for a parameter that are biologically feasible and are assigned to represent values of the mechanism for which that parameter represents. Values (and ranges) are assigned according to biological observations, experimental results, or mathematical estimation.

**Parameter Space:** The set of all combinations of parameter values for a particular model, as defined by the parameter ranges for each parameter.

**Uncertainty and Sensitivity Analysis:** A series of techniques used to evaluate the influence a parameter has on model outcomes. Influence of individual mechanism can be assessed (see Methods for more details).

**Calibration:** The process of varying parameters until the model behavior reaches a preferred end state or predetermined goal (usually the dataspace).

**Initial Conditions:** The predefined initial values of each variable in a mathematical model prior to simulating the model. In this work, initial conditions were also varied during model calibration as initial condition could represent pre-existing immune memory cells.

**Radar Charts:** A graphical visualization of multivariate data across multiple axis. We use radar charts to display the parameter space of our simulations.

We quantify the importance of each host mechanism involved in vaccination dynamics by finding correlations between model parameters and outputs. Correlations between specific model outputs and parameters were determined by using Partial Rank Correlation Coefficient (PRCC), where−1 denotes a perfect negative correlation between a model output and parameter (+1 denotes a perfect positive correlation between model output and parameter). Marino et al. completed a review of the statistical tests available to access significance of PRCC (Marino et al., 2008). PRCC results performed a dual role: not only do they reveal the relationship between model outcomes and parameters, they also inform calibration of the model to the immunogenicity dataspace. As the model is tuned, manipulations to the more sensitive parameters ameliorate model fitting according to the criteria above.

Since our model provides measurements in the form of cell counts in lymph node and cells/mm∧3 in the blood, we performed post-processing of the simulations to ensure that units matched those provided by the H56 vaccination data (See Supplementary Materials 1 for details).

### Parameter space visualization

We utilized radar charts to illustrate parameter range comparisons between species and the impact of BCG on cellular responses. Radar charts are a graphical visualization of multivariate data across multiple axis. In this work, we plotted radar charts using *R's* radarchart function in the fmsb package (Nakazawa, 2017). Each axis represents a parameter of interest in our ODE model. Points near the center of each axis represent a lower value for that parameter whereas points near the outer edges of each axis represent larger values. To compare parameter ranges across species, we calculate the minimum and maximum for each axis on the charts as the minimum and maximum value for each parameter across all species and antigen-specific fits (see Supplementary Materials 2). To compare the impact of BCG memory on the H56 immune response, we created the human radar charts with a minimum and maximum for each axis defined by the minimum and maximum parameter value across human model fits to ESAT6 or Ag85B. We created the NHP radar charts by displaying the parameter ranges within the minimum and maximum values across NHP model fits to either antigen.

## Results

### Humans and non-human primates exhibit different T-cell responses to ESAT6 following H56 vaccination

**Figure 3:**
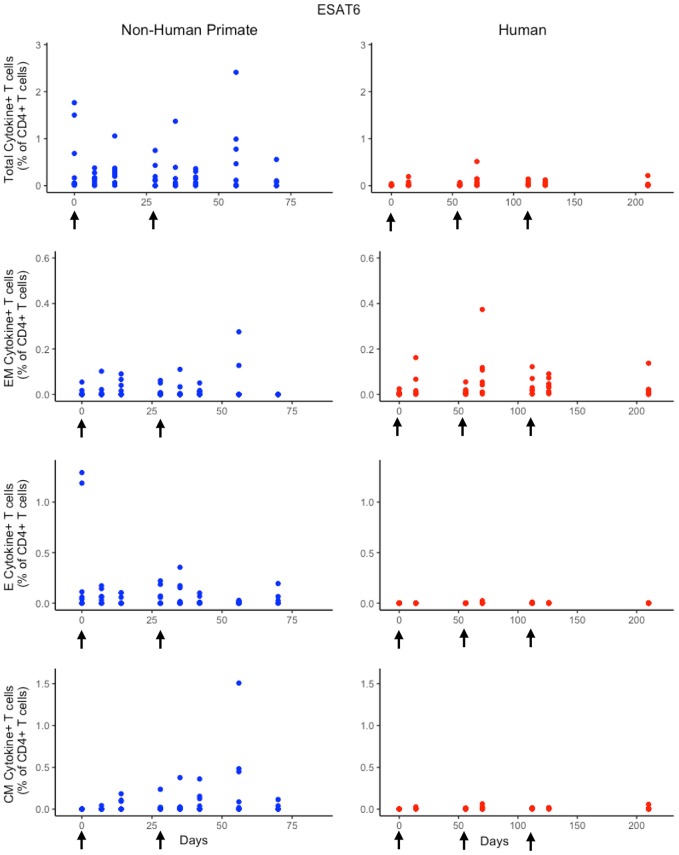
Experimental data show different responses to ESAT6 antigen following H56 vaccination. The percentage of blood CD4+ T cells that respond to ESAT6 by producing cytokines (cytokine+) is divided by the total number of CD4+ T cells in the blood. T cell subtypes are also shown. Each time point shows the responses of all 8 human (red) or all 8 NHPs (blue) subjects. Note that it can be difficult to perceive 8 individual dots–if the subject's responses are similar or the same, as individual dots overlap. For ease of comparison, we have placed both panels of data on the same y-axis. Arrows represent vaccination timepoints.

In response to H56 vaccination, humans and NHPs showed large variability within and across species. While some of this variability can be attributed to the different experimental protocols used (Figure 1), the magnitudes of responses between species still differ. Several differences in the magnitude and timing of response across species are notable (Figure 3). The total response of CD4+ ESAT6+ T cells in NHPs is larger and more variable than the response in humans. For example, an *F* test to compare variances between the two species at day 14 reveals a significant difference (*p* = 0.0003; variance of NHPs was approximately 25 times greater than the humans). Day 14 is the final day that protocols follow the same timelines. Therefore we selected day 14 for this statistical test in order to exclude variability due to different experimental protocols.

Furthermore, the magnitude of effector and central memory population responses is larger in NHPs than humans. Between species, the effector memory subpopulation responses are most similar. The major contributors to the total NHP CD4+ ESAT6+ T-cell response are the effector T cell population during early timepoints and the central memory T cell population at later timepoints. The human response is dominated by effector memory T cells. Interestingly, some data suggest that the dose of H56 used in this study may also have contributed to this exaggerated memory T cell response; current thinking will pursue at least a 10-fold lower dose.

### A single mathematical model describes both human and NHP T cell responses to ESAT6

**Figure 4:**
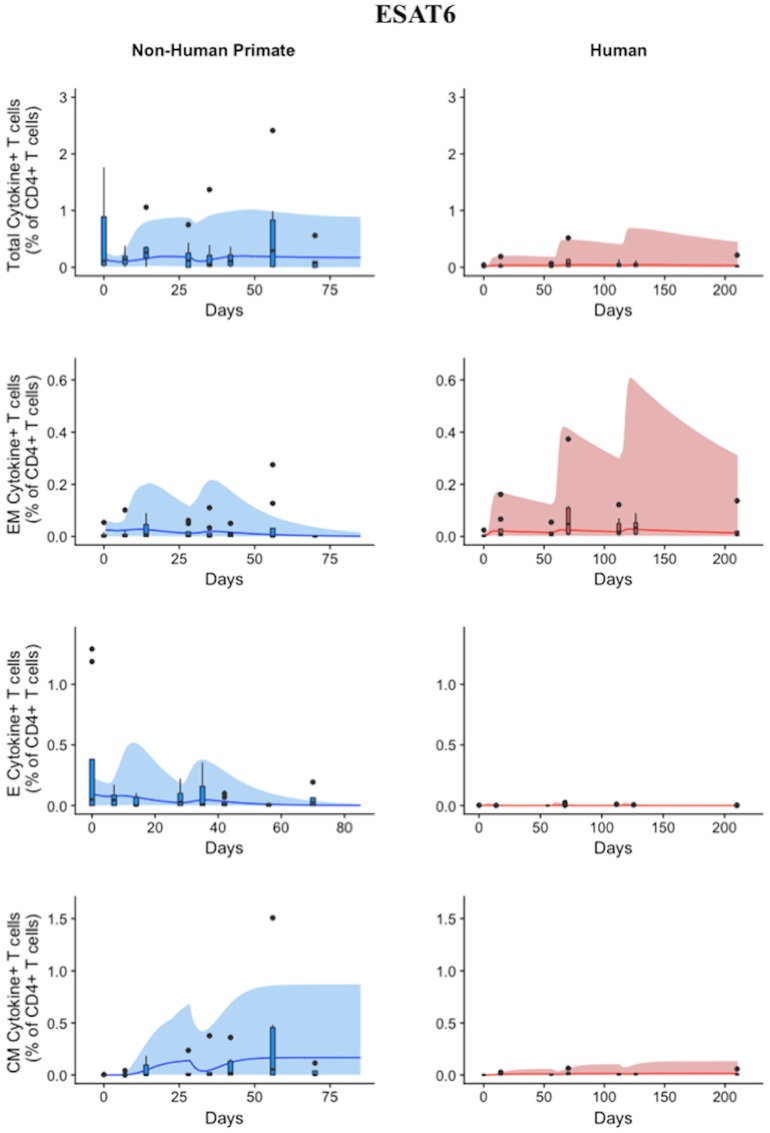
Model captures diverse response of both NHP and Humans to ESAT6 antigen following H56 vaccination. The percentage of blood CD4+ T cells that respond to ESAT6 by producing cytokines (cytokine+) is divided by the total number of CD4+ T cells in the blood. T cell subtypes are also shown. Each time point shows the responses of all 8 human (red) or all 8 NHPs (blue) subjects using a box and whisker plot. These box and whisker plots provided a guide for the boundaries of immunogenicity dataspace. Whiskers were created by extending from the edge of the box to the data point that is the closest, but does not exceed 1.5 times the interquartile range (defined as the distance between the first and third quartiles) from the edge of the box. Any experimental points beyond the edge of the whisker are deemed as outliers and plotted as black points. Simulation data are displayed as a blue or red cloud that outline the min and max of 500 runs for NHP or human calibrations, respectively. The blue or red line represents the median of those simulations. Our goal when calibrating to cell levels in blood of both species was to ensure that *in silico* simulations fell reasonably within these dataspaces, as outlined in the Methods section. Parameter ranges used to generate the simulation curves are shown in Supplementary Table 1.

Statistically, we have shown that there is a difference in NHP and human responses to ESAT6. However, statistical analysis could not answer the following questions: (1) Are the data for both humans and NHPs consistent with the same mechanisms for mounting an immune response? (2) If those mechanisms are the same, can the rates of proliferation and differentiation alone be responsible for the differences we observe in ESAT immunogenicity? These questions require a method that can address the dynamics of priming, proliferation, and differentiation that are intrinsic to the development of an immune response. In Methods, we present a mathematical model that describes T cell priming, proliferation, and differentiation in response to APCs in the blood and LN of primates. Here, we hypothesize that this mathematical model can capture both human and NHP T cell responses to ESAT6; however, it will require the use of different sets of parameter values. In Figure 4, experimental data from Figure 3 were replotted as box and whisker plots (blue–NHP, red–human) and simulation curves are shown by the cloud and median lines (blue and red, respectively).

NHP simulation data recapitulates the variability in the experimental data by capturing the dynamics of the experimental data. In particular, the median simulation line demonstrates how the model captures the general behavior of the data, by traveling through the interquartile range of at least 4 of the 8 timepoints for each subpopulation of T cells. The human simulations capture the clinical data—our maximum and minimum simulations include nearly all of the outlying data points across the subpopulations of T cells. A visual comparison of these parameter ranges is displayed in Supplementary Materials 2. Altogether, we demonstrate that our model captures the ESAT6 immunogenicity dataspace of both NHPs and humans—suggesting that the mechanisms of generating a primary immune response are the same for both NHPs and humans.

### Sensitivity analysis reveals both similar and distinct outcome drivers across species in response to ESAT6

Having calibrated our model to both ESAT6 human and ESAT6 NHP immunogenicity dataspaces, we next used these two model fits to ask questions about important processes within the CD4+ T cell response. In particular, we wanted to better understand the dual roles of proliferation and differentiation that drive immune response magnitude and timing following vaccination in both species. To investigate these processes, we performed uncertainty and sensitivity analysis on 3 outcomes (ESAT6-specific central memory, effector, and effector memory T cell subtypes) of our model. Table 1 highlights processes (i.e., parameters) found to be significantly associated with changes in T cell response subpopulations for each species.

**Table 1:** Parameters with significant PRCCs for ESAT6 immune response outcomes.

**ESAT6**	**Central memory**	**Effector**	**Effector memory**
NHP	central memory reactivation rate; precursor proliferation and differentiation into central memory cells; APC and precursor death rates	precursor proliferation and differentiation into effector cells; effector, APC, and precursor death rates	precursor proliferation and differentiation into effector cells; APC and precursor death rates
Human	Likelihood of proliferation; precursor proliferation and differentiation to central memory; central memory recruitment; APC, and precursor death rates;	Likelihood of proliferation and differentiation; Naïve T cell recruitment; Precursor proliferation and differentiation to Effector; effector differentiation to effector Memory; effector Lymph efflux; effector, APC, and precursor death rates;	Likelihood of proliferation and differentiation; precursor proliferation; effector memory, APC, and precursor death rates;

*One row displays humans, the other displays NHPs. Columns list the 3 model outcomes of interest – ESAT6-specific central memory, effector and effector memory T cell phenotypes. These outcomes were selected for analysis because the model was calibrated to their dataspace. Each table cell contains a general description of significant (i.e., p < 10^−3^) parameters with respect to each output of the model*.

For both species, uncertainty and sensitivity analysis support a key role for priming and proliferation within lymph nodes. This is not a novel concept, but rather acts as a proper control for the utility of our model, as it is accepted that priming and proliferation within the lymph node underlies immunogenicity of a vaccine (Moliva et al., 2017). Specifically, uncertainty and sensitivity analysis revealed a crucial role for CD4+ T cell precursor proliferation rates (k4) within the lymph node compartment. The significant, positive association between precursor T cell proliferation rates and 3 different T cell subtypes in the blood represents an inter-compartmental effect–not only does the parameter influence the dynamics within its own compartment (lymph node), it drives the dynamics of the compartment yielding experimentally validated results (blood).

There were also modest differences in the mechanisms driving model fits for NHP and humans, (Table 1). For example, only the human dataset showed significant negative correlations between cellular responses in the blood and the half-saturation values of precursor proliferation and differentiation in the lymph node (represented as “likelihood of proliferation and differentiation” in Table 1). We predict that humans and NHPs are generally alike in response to ESAT6, but proliferation and differentiation in humans is not quite as easily triggered as proliferation and differentiation in the NHP. This could be in part due to the influence of humans regularly exposed to many and diverse environmental factors.

### Humans and non-human primates exhibit different T-Cell responses to Ag85B following H56 vaccination

**Figure 5:**
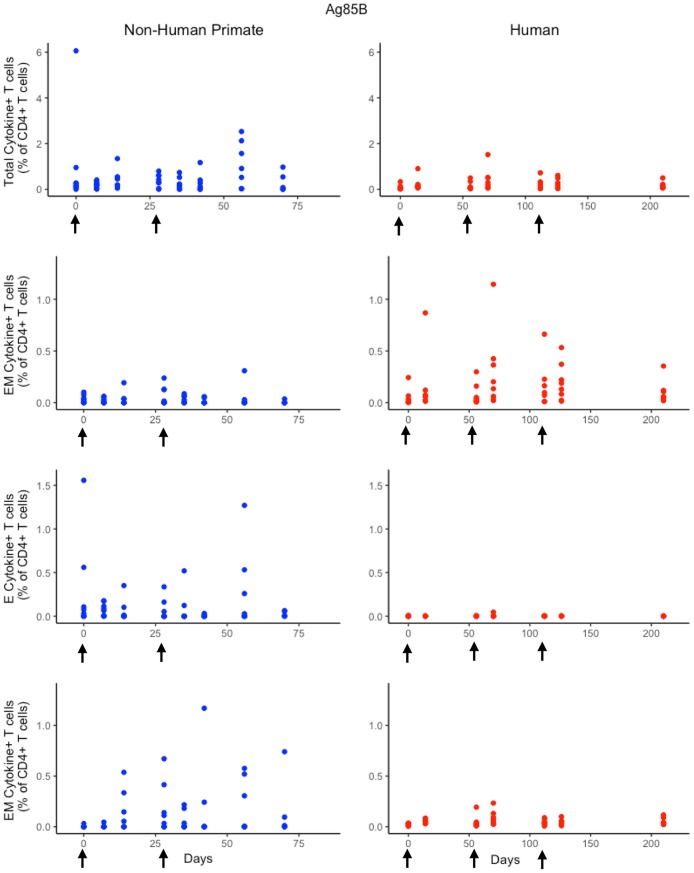
Human and NHP experimental data show different responses to Ag85B antigen following H56 vaccination. The percentage of blood CD4+ T cells that respond to Ag85B by producing cytokines (cytokine+) is divided by the total number of CD4+ T cells in the blood. T cell subtypes are also shown. Each time point shows the responses of all 8 human (red) or all 8 NHPs (blue) subjects (some responses overlap, so it might be difficult to see 8 distinct dots). For comparison, we placed both panels of data on the same y-axis. Arrows represent vaccination timepoints.

While the immunological response between humans and NHPs to the ESAT6 antigen in H56 vaccination can be attributed to intrinsic similarities and differences between species, the response to the Ag85B antigen offers an opportunity to investigate the role of prior BCG vaccination on H56 immunogenicity (Figure 5). When we compare magnitude and timing of responses across species, several differences emerge. As observed for responses to ESAT6, the total response of CD4+ Ag85B+ T cells in NHPs is higher and more variable than the response in humans. For example, an *F* test to compare variances for the central memory T cell population at day 14 revealed a significant difference (*p* = 3.984e-06; variance in NHPs is about 96 times greater than humans). While the magnitude of effector and central memory subpopulation responses were larger in NHPs, it appeared that humans had a larger effector memory subpopulation response.

### A single mathematical model describes NHP and human T cell responses to Ag85B

**Figure 6:**
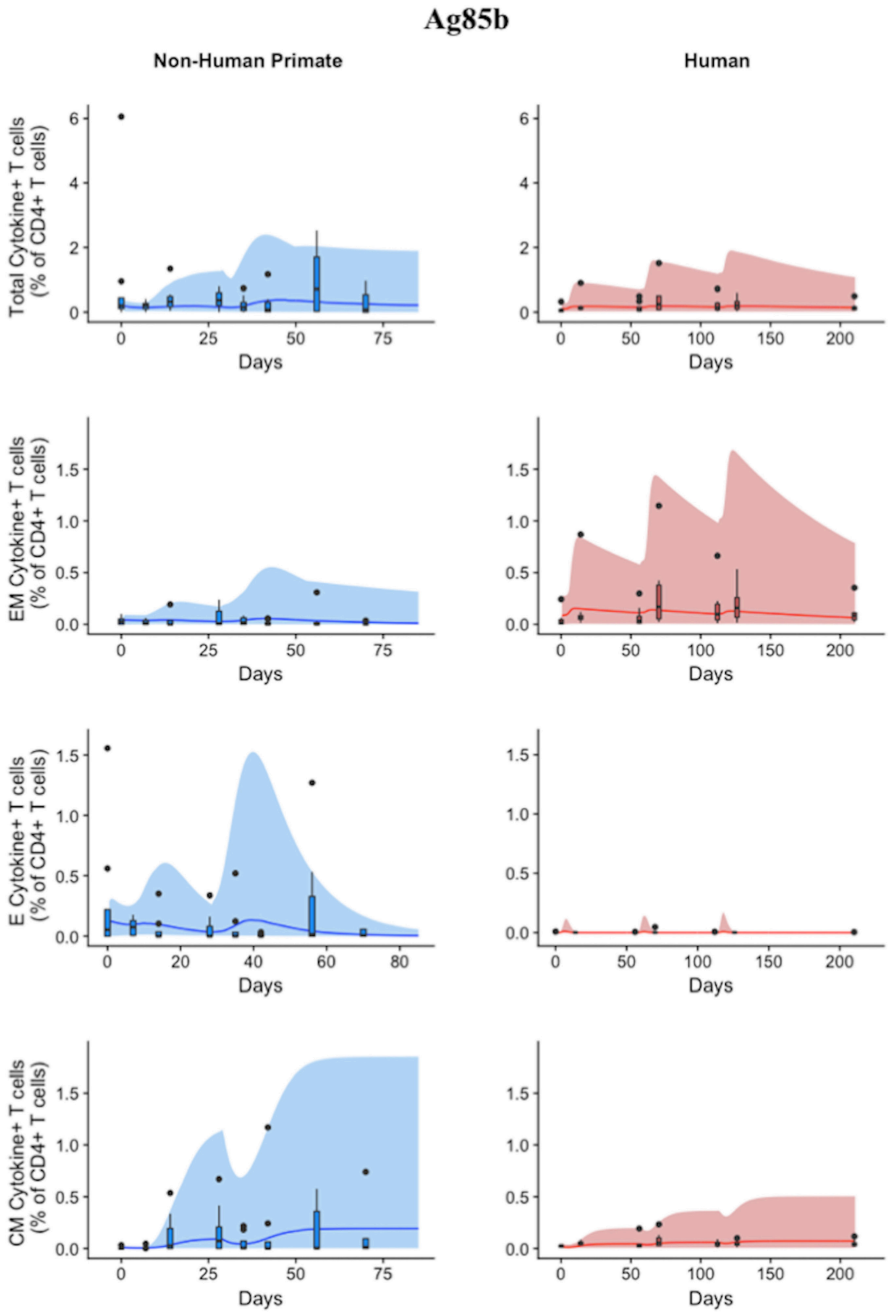
Model can fit diverse responses of both NHP and Humans to Ag85B antigen following H56 vaccination. The percentage of blood CD4+ T cells that respond to Ag85B by producing cytokines (cytokine+) is divided by the total number of CD4+ T cells in the blood. T cell subtypes are also shown. Each time point shows the responses of all 8 human (red) or all 8 NHPs (blue) subjects as a box and whisker plot. Whiskers were created in the same manner as the ESAT6 datasets. Simulation data are displayed as a blue or red cloud that outline the min and max of 500 runs for NHP or human calibrations, respectively. The blue or red line represents the median of those simulations and demonstrates that the model captures the general behavior of the data, by traveling through the interquartile range of at least 4 of the 8 timepoints for each subpopulation of T cells. Exact parameters ranges used to generate the simulation curves for NHP and human CD4+Ag85B+ T cells are shown in Supplementary Table 1.

Using statistical analysis, we have revealed a difference between species in immune response to Ag85B. However, statistical analysis cannot answer the following questions: (1) what is the impact of different BCG timing on H56 response? (2) is the influence of BCG prime on H56 immune response the same for both species—i.e., do the two species possess a similar secondary response to an antigen? To mechanistically understand the role and timing of BCG prime on H56 vaccination, we require a mathematical modeling approach to predict dynamics of the different T cell responses to Ag85B. As with ESAT6, we tested whether our mathematical model can capture the Ag85B immunogenicity dataspace for both NHPs and humans (Figure 6). Our simulation data mimic the variability in the NHP experimental data by tracking most outlier points and whiskers. For example, simulations reflect a contraction of the central memory population and follow expected logic—a percentage of central memory cell populations will reactivate and become precursor T cells in the LN. Thus, the percentage of central memory T cells should contract within blood.

The human simulations also capture the variability of the human dataset as well as the general trends, as shown by the median red line. A visual comparison between the parameter ranges is displayed in Supplementary Materials 2 using radar charts. Altogether, we show that our mathematical model can capture the Ag85B immunogenicity dataspace of NHPs and humans with species-specific parameter ranges.

### Differences in BCG timing between humans and NHPs is captured by initial conditions

Throughout our calibration process, we were aware of the potential for the timing of BCG priming events to influence theimmune response of each species to Ag85B (as NHPs received BCG vaccination 70 days before H56 vaccination and humans received their BCG vaccination roughly 30 years before the clinical trial began – see Methods and Figure 1). Instead of explicitly modeling a BCG vaccination event 70 days or 30 years prior to H56 vaccination, we varied initial concentrations of memory cell types in the LN and blood as a proxy for these BCG vaccinations. The initial cell concentrations represent the value of memory antigen-specific T cells within the system. That is, these T cells, prior to vaccination with H56, were specific for the Ag85B antigen. The initial condition values that led to the best model fits for both NHP and human T cell response are shown in Table 2. Note that the abbreviated time between BCG and H56 vaccinations for NHPs meant that many precursor CD4+ T cells were present in the LN; this population may well have waned in humans who were vaccinated many years (to decades) prior. As a portion of these precursor T cells differentiate into central memory T cells and effector T cells, the BCG vaccination event enabled the model to recapitulate the immunogenicity dataspaces for these two T cell subpopulations and could also explain the larger NHP response to the vaccine.

**Table 2:** Initial conditions represent the difference in BCG timing between experimental protocols.

**Initial condition of cell type**		**ESAT6**			**Ag85b**		
		**NHP**		**Human**	**NHP**		**Human**
	**Units**	**Range of values**		**Range of values**	**Range of values**		**Range of values**
Naïve CD4+ specific Blood T cells	cell/mm^3^	(0.1,0.25)		(0.07, 0.6)	(0.17,0.37)		(0.04,0.27)
Effector CD4+ specific Blood T cells	cell/mm^3^	(0.001,1.5)		0	(0.001,2.5)		0
Central Memory CD4+ specific Blood T cells	cell/mm^3^	(0.0015,0.006)		(0.00002, 0.03)	(0.002,0.2)		(0.02,0.3)
Effector Memory CD4+ specific Blood T cells	cell/mm^3^	(0.001,0.5)		(0.003, 0.15)	(0.003, 0.9)		(0.0016,2.6)
Naïve CD4+ nonspecific Blood T cells	cell/mm^3^	(160,240)		(100,600)	(241,361)		(59,272)
Effector CD4+ nonspecific Blood T cells	cell/mm^3^	(200,800)		(530,110)	(445, 670)		(358,875)
Central Memory CD4+ nonspecific Blood T cells	cell/mm^3^	(1,3)		(0.009,10)	(1,100)		(10,100)
Effector Memory CD4+ nonspecific Blood T cells	cell/mm^3^	(1,150)		(1,22)	(1,300)		(0.3,370)
Naïve CD4+ specific LN T cells	cell count	(91957, 322492)		(8255,111806)	(144500,546200)		(5000,5720)
Precursor CD4+ specific LN T cells	cell count	0		0	(6770, 10150)		0
Effector CD4+ specific LN T cells	cell count	0		0	(22,34)		0
Central Memory CD4+ specific LN T cells	cell count	(1295,7878)		(3.4, 5046)	(2377,285871)		(3132, 59431)
Effector Memory CD4+ specific LN T cells	cell count	0		0	(828,1241)		0
Naïve CD4+ nonspecific LN T cells	cell count	(123430594,355639025)		(11839508, 122029962)	(177300481, 535316901)		(7865162, 53811216)
Central Memory CD4+ nonspecific LN T cells	cell count	(775507,4253381)		(1229, 1895598)	(1219316, 134489106)		(1401106, 19893946)
APC (Prime Vaccination of H56)	cell count	(150,800)		(200,500)	(350,500)		(500,1000)
APC (Boost Vaccination 1 of H56)	cell count	(50, 150)		(200,500)	(250,500)		(400,600)
APC (Boost Vaccination 2 of H56)	cell count	*****	*****	(200,500)	*****	*****	(400,600)

*The disparity between initial condition values that preceded the NHP response and those corresponding values for the human response represent the impact of prior presentation of Ag85B via BCG on the system*.

***** *signifies that NHP experimental protocol did not give the NHPs a second boost of H56 vaccination*.

### Sensitivity analysis reveals both similar and distinct outcome drivers across species in magnitude of T-cell responses to Ag85B antigen

We performed uncertainty and sensitivity analysis on the same 3 model outcomes as the ESAT6 response analysis to identify important processes in CD4+ T cell response to Ag85B in each species. We identified factors, such as CD4+ central memory cell recruitment, to be significantly associated with changes in T cell response subpopulations (Table 3). Uncertainty and sensitivity analysis also revealed a crucial role for CD4+ Precursor proliferation and half-saturation rates within the lymph node compartment (Table 3).

**Table 3:** Significant PRCCs for Ag85B immune response outcomes.

**Ag85B**	**Central memory**	**Effector**	**Effector memory**
NHP	central memory reactivation rate; Likelihood of differentiation; precursor proliferation and differentiation into central memory cells; APC and precursor death rates	Likelihood of differentiation; precursor proliferation and differentiation into effector cells; effector, APC, and precursor death rates	precursor proliferation and differentiation into effector cells; APC and precursor death rates
Human	Likelihood of proliferation; precursor proliferation and differentiation into central memory; central memory recruitment rate; APC and precursor death rates	Likelihood of proliferation and differentiation; naïve T cell recruitment; precursor proliferation and differentiation to effector; effector differentiation to effector memory; effector Lymph efflux; effector, APC, and precursor death rates	Likelihood of proliferation; precursor Proliferation; effector memory, APC, and precursor death rates

*One row represents humans, the other represents NHPs. Columns list the 3 model outcomes of interest–Ag85B-specific central memory, effector and effector memory T cell phenotypes. These outcomes were selected for analysis because the model was calibrated to their dataspace. Each table cell contains a general description of significant (i.e., p < 10^−3^) parameters with respect to outputs of the model*.

Modest differences also exist in the mechanisms driving model fits for NHP and human (see Supplementary Table 1). In addition to the stark differences in initial conditions (from BCG timing), uncertainty and sensitivity analysis predicts that in NHPs, central memory reactivation rates were significantly associated with the total CD4+Ag85B+ response outcome. The importance of reactivation in the central memory population supports not only the role of BCG memory in this system, but could indirectly explain the late increase in Ag85B+ effector cells around day 56 (as the central memory cells that reactivate become precursor cells that, in turn, can become effector cells). Overall, the human and NHP Ag85B responses differ in values of initial conditions, central memory reactivation, and T cell differentiation. Despite these differences, like the ESAT6 response, we predict that the Ag85B response in NHPs and humans are generally alike–this similarity hints at a general secondary response that is conserved across species.

### Secondary response to Ag85B antigen is characterized by the upregulation of differentiation to central memory phenotype

**Figure 7:**
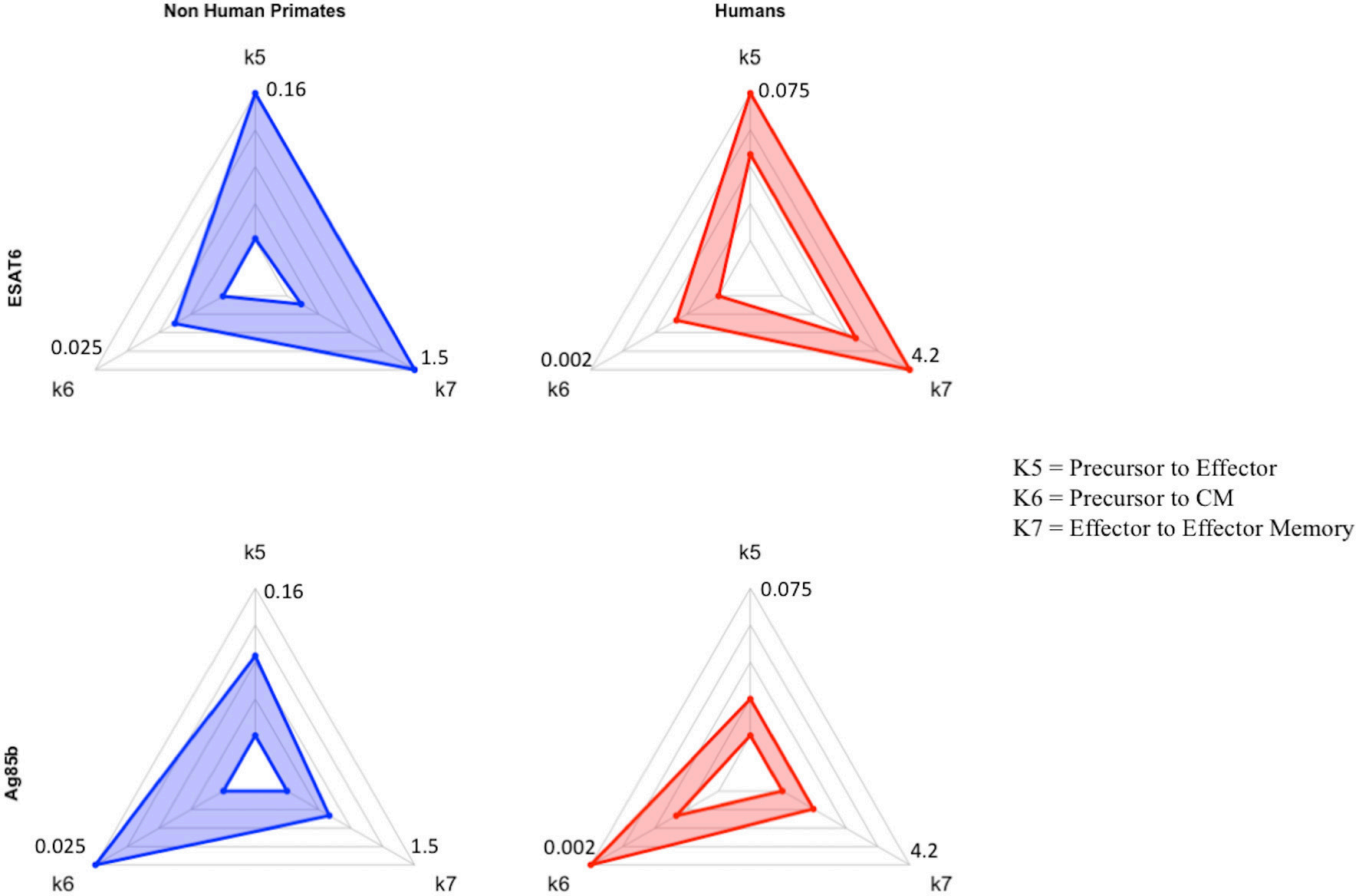
Radar charts reveal impact of immunological memory in response to Ag85B. We display 4 radar charts (see Supplementary Materials 2 and Methods) that visually represent the parameter space for several key parameters (as identified by PRCC) in model fits for both species and antigens. Each chart includes the maximum value of each parameter (for each species) on the diagrams. The top chart shows the parameter ranges that fit the ESAT6 immunogenicity dataspace. The bottom radar chart displays the parameter ranges that fit the Ag85B immunogenicity dataspace. These parameters were picked as they represent T-cell differentiation rates to central memory (k6), effector (k5), and effector memory (k7) T cell phenotypes. Each parameter space is represented by a blue (NHP) or red (human) band, which represents the min and max parameter value for each model fit. Supplementary Table 1 shows the numerical values of the parameter ranges. To directly compare the impact of BCG memory on the H56 immune response, we created the Human radar charts with a minimum and maximum for each axis defined by the minimum and maximum parameter value across Human model fits to ESAT6 or Ag85B. We created the NHP radar charts by displaying the parameter ranges within the min and maximum values across NHP model fits to either antigen. Viewers should not compare the charts from left to right, as the human charts display a parameter range that is wholly distinct from that of the non-human primates. For each species, the maximum values for each parameter are displayed at the edges of the radar charts.

If we consider the T cell response of NHP and humans to ESAT6 as the epitome of each species' primary response to an antigen in vaccination, then we can view the parameter values that recapitulate the Ag85B response (a secondary response to the same antigen) as a BCG-induced modification to the parameter values that captured the ESAT6 response. For NHPs (blue) and humans (red), three parameters (k5, k6, k7) are represented on each axis of the radar charts for ESAT6 and Ag85B (Figure 7). Notice that, for each species, the radar charts include the maximum value for each parameter across the ESAT6 and Ag85B response fits. In the ESAT6 radar charts, both NHPs and humans skew toward the differentiation of effector and effector memory T cell phenotypes. As neither species has encountered ESAT6 prior to H56 vaccination, the relatively high rates of differentiation to effector and effector memory T cell phenotypes constitute a primary response that may be conserved across species.

Ag85B is an antigen that was first presented in BCG vaccination; if we compare the dynamics of ESAT6 responses to the dynamics of Ag85B responses, we can predict the BCG-induced modifications to T-cell differentiation during secondary responses to the same antigen. In the Ag85B radar charts, both species' ranges for differentiation to effector and effector memory become relatively smaller than the ranges that fit the ESAT6 response. Further, the ranges for the parameter that captures differentiation to a central memory phenotype grow larger relative to the ranges shown in ESAT6 response. We speculate that this change in response is conserved across species – upon secondary response to the same antigen, both species' precursor T-cell populations upregulate the production of a central memory phenotype during differentiation.

## Discussion

In the pursuit of a vaccine that can confer long-term, consistent immunity against TB, H56 is one new vaccine candidate. However, the role of prior BCG vaccination on H56 immunogenicity is unclear. In addition, the differences between NHP–a useful model animal for vaccine studies - and human responses to H56 has not been explicitly characterized. Identifying the influence of BCG on H56 vaccination and characterizing the species-specific responses to H56 will better facilitate our understanding of H56 immunogenicity and could potentially pave the way for more effective therapies. In addition, we strive to elaborate how computational modeling can assist with vaccine development and testing.

In this work, we used a systems biology approach that utilized mathematical modeling to explore both NHP and human response datasets to H56. We calibrated our two-compartment mathematical model to the ESAT6 and Ag85B immunogenicity dataspaces for both NHPs and humans. This calibration allowed us to study pre-exposure vaccination dynamics such as antigen presentation, T cell priming, and differentiation in both the lymph node and blood. Specifically, we utilized antigen specificity to draw our main conclusion: *BCG similarly influences H56 immunogenicity in both NHPs and humans* by upregulating differentiation to the central memory phenotype in the Ag85B-specific CD4+ T cell response. While Lin *et al*. found that H56 boosts the effects of BCG and prevents reactivation of latent infection (Lin et al., 2012), to our knowledge no one has documented the direct impact of prior BCG on H56 immunogenicity.

Using mathematical modeling, we were also able to isolate the impact of BCG timing differences on H56 immunogenicity. We discovered that the narrow window between BCG prime and H56 vaccination in NHPs promotes a larger quantity of antigen-specific cells that reside in the lymph node prior to H56 vaccination. Calibration to the Ag85B immunogenicity dataspace for NHPs revealed a much larger initial number of precursor T cells in the lymph node than the number of initial precursor cells that were required for calibration to the human data. The difference in timing of BCG for the NHP experimental protocol (70 days prior to H56 vaccination) and human experimental protocol (up to decades before H56) explains the necessary differences required in model initial conditions to capture these events. Experimental assessment of vaccines in NHPs preclude the administration of BCG years prior to boosting with a subunit vaccine, due to costs. However, our data indicate that the timing of BCG and booster vaccines strongly influence the subsequent immune responses. Whether this also affects protection conferred by a vaccine remains to be tested.

Using uncertainty and sensitivity analysis, we found that each species' response to H56 vaccination was generally similar. While each species resides in a separate parameter space, the general dynamics dictating the H56 immune response was quite similar. This finding contrasts with previous findings that show the immune response of monkeys and humans to SIV or HIV (respectively) differs (Davenport et al., 2004; Yang and Ganusov, 2017), however, like many others in the field of TB research, we conclude that NHPs are a good model for human responses (Kaushal et al., 2012; Scanga and Flynn, 2014; Flynn et al., 2015; Peña and Ho, 2015). However, one consistent difference between NHP and human response were observed. Unlike the NHP response, the humans' central memory, effector, and effector memory T cell phenotypes was significantly negatively correlated with the half-saturation values of proliferation and differentiation in both the ESAT6 and Ag85B immunogenicity dataspaces. As the half-saturation values in our model measure the affinity (or likelihood) of a cell to proliferate or differentiate upon priming, our findings suggest that humans differ from NHPs in the ability of T cells to quickly react to H56 vaccination antigens within lymph nodes. Perhaps presentation of these antigens to T cells is not as effective in humans as it is in NHPs. We indirectly modeled adjuvant impact on vaccination (see Methods); however, a more mechanistic approach may be necessary to elucidate these species-specific differences in antigen uptake and presentation.

Furthermore, uncertainty and sensitivity analysis revealed an intriguing result regarding the human experimental protocol. Throughout our analysis, the number of APCs that entered the system via vaccinations (prime or boost events) was significantly, positively, associated with cellular responses in the blood. However, our analysis also showed that the number of APCs that entered the system as a result of the second boosting event (third H56 vaccination event) for humans did not significantly impact the number of central memory T cells within the blood compartment. This result agrees with the previous finding that 50 ug of H56 is too high of a dose (Luabeya et al., 2015), resulting in large effector responses that may be suboptimal for long-term memory. As one major goal of any vaccination is to provide long lasting immunity in the form of immunological memory, our computational analysis has revealed that the third dose was likely redundant and that optimization of dose using computational predictions could have potentially improved outcomes, especially prior to the clinical trial. In the future our systems biology approach together with virtual clinical trials could help investigate these issues and assist in improving the vaccine pipeline.

One potential limitation of this study is that our current model represents the complex processes of proliferation, differentiation, and reactivation rates as a single parameter with a range of values. We believe this suffices since our goal was to identify the role of BCG in H56 vaccination response across humans and NHPs. However, future investigations into the processes dictating proliferation, differentiation, or reactivation could create a more detailed mathematical model including those details. In fact, the field of T-cell memory and the exact mechanisms of reactivation have been extensively studied (Harrington et al., 2008; MacLeod et al., 2010; Akondy et al., 2017; Youngblood et al., 2017). Conversely, phenomenological modeling has provided insights for T cell expansion (Davenport et al., 2004; Antia et al., 2005; Akondy et al., 2015). Future work could discuss the benefits of mechanistic or phenomenological models when addressing distinct questions about proliferation, differentiation, or reactivation.

In summary, we used a systems biology approach that combined NHP and human datasets with mathematical modeling to better understand the differences between NHP and human immune response to H56 vaccination. Specifically, we showed that each primate species had a similar response to H56, identified the role of BCG timing on H56 vaccination, and discovered that BCG similarly influences H56 immunogenicity in humans and NHPs.

Beyond the scope of this paper, we could have characterized other comparisons between humans and NHPs. For example, future studies could identify the species-specific differences during TB infection, identify the adaptive immune response differences to other antigens, or capture the dissimilarities of each species' innate immune response to adjuvant. Further, future studies could also model the cellular dynamics following H56 vaccination before, during, or after TB infection in an effort to evaluate the potential success of this vaccine candidate. We argue that a systems biology approach that melds mathematical modeling together with experimental and clinical studies has the greatest potential to discover, predict, and evaluate new vaccination strategies that could end the TB epidemic.

## Author contributions

LJ, EP, JL, and DK performed mathematical modeling, data analysis. LJ, EP, JL, DK, TS, and JF contributed conception and design of the study. RD and JF prepared and sorted NHP dataset. SS, BK, and TS prepared and sorted the human H56 dataset. All authors contributed to writing and editing of this manuscript.

### Conflict of interest statement

The authors declare that the research was conducted in the absence of any commercial or financial relationships that could be construed as a potential conflict of interest.

## Supplementary material

Supplementary Figure 1 Radar charts reveal parameter space differences between species. Each parameter space is represented by a blue (NHP) or red (human) band, which represents the min and max parameter value for each model fit. Each chart displays parameter names around its outside boundary, at each axis. Parameter names are ordered alphabetically starting with ‘hs1’ and ‘ending with xi6’. Points near the center of each axis represent a lower value whereas points near the outer edges of each axis represent larger values. To compare parameter ranges across species, we calculated the minimum and maximum for each axis on the charts as the minimum and maximum value for each parameter across all species and antigen specific fits.

Supplementary Table 1 Parameter ranges for model fits of the ESAT6 and Ag85B response in humans and NHPs. Parameter names, descriptions, units, and ranges are listed.

Supplementary Table 2 Parameter names in radar charts. The leftmost column shows the name of each parameter. The rightmost column displays a short description of each parameter.
