# Supplementary material for: Integrating Non-human Primate, Human, and Mathematical Studies to Determine the Influence of BCG Timing on H56 Vaccine Outcomes
Source: Front Microbiol. 2018 Aug 17;9:1734. doi: 10.3389/fmicb.2018.01734 (PMC6109686; doi:10.3389/fmicb.2018.01734)
Supplement: Supplementary Figure 1 — Radar charts reveal parameter space differences between species. Each parameter space is represented by a blue (NHP) or red (human) band, which represents the min and max parameter value for each model fit. Each chart displays parameter names around its outside boundary, at each axis. Parameter names are ordered alphabetically starting with ‘hs1’ and ‘ending with xi6’. Points near the center of each axis represent a lower value whereas points near the outer edges of each axis represent larger values. To compare parameter ranges across species, we calculated the minimum and maximum for each axis on the charts as the minimum and maximum value for each parameter across all species and antigen specific fits. [file Data_Sheet_2.DOCX]

Supplementary Material 2

Visual comparison of parameter ranges for simulation curves

Louis Joslyn, Elsje Pienaar, Robert M. DiFazio, Sara Suliman, Benjamin M. Kagina, JoAnne L. Flynn, Thomas J. Scriba, Jennifer J. Linderman^*^, Denise E. Kirschner^*^

*** Correspondence:**

Denise E. Kirschner: kirschne@umich.edu , Jennifer J. Linderman: linderma@umich.edu

# Despite similar drivers across species, non-human primates and humans exist in separate parameter spaces

This supplementary text will present the parameter ranges for human and NHP ESAT6 and Ag85b immunogenicity dataspaces. Supplementary Figure 1 shows the radarcharts that visually display the parameter ranges. Radarchart construction is outlined in the Methods section of the main paper. Briefly, this chart is to be read from left to right. That is, the charts are constructed such that in the ESAT6 (or Ag85b) row, we can compare the parameter ranges that fit the NHP response (blue) to those that fit the Human response (red). A small table is shown beneath the figure, to provide the reader with a legend for parameter names.

While sensitivity and uncertainty analysis (discussed in main paper) reveals the parameters that generally control responses to each antigen, it is important to broadly consider the differential parameter spaces whereupon these responses reside. Uncertainty and sensitivity analysis suggested that parameters for Precursor cell proliferation (k4), differentiation (k5 and k6), recruitment rates of Naïve (k1) and Central Memory (k8), and half-saturation values (hs1-hs8) all played important roles in at least one of the three main blood T cell subtype outcomes. However, examination of Supplementary Figure 1’s charts allows for direct comparison of each species’ parameter ranges for each of these parameters. These charts reveal that each species resides in a separate parameter space for the majority of these parameters. Thus, despite the similar (revealed by uncertainty and sensitivity analysis) processes of each species, the parameters that dictate these processes actually differ in value.


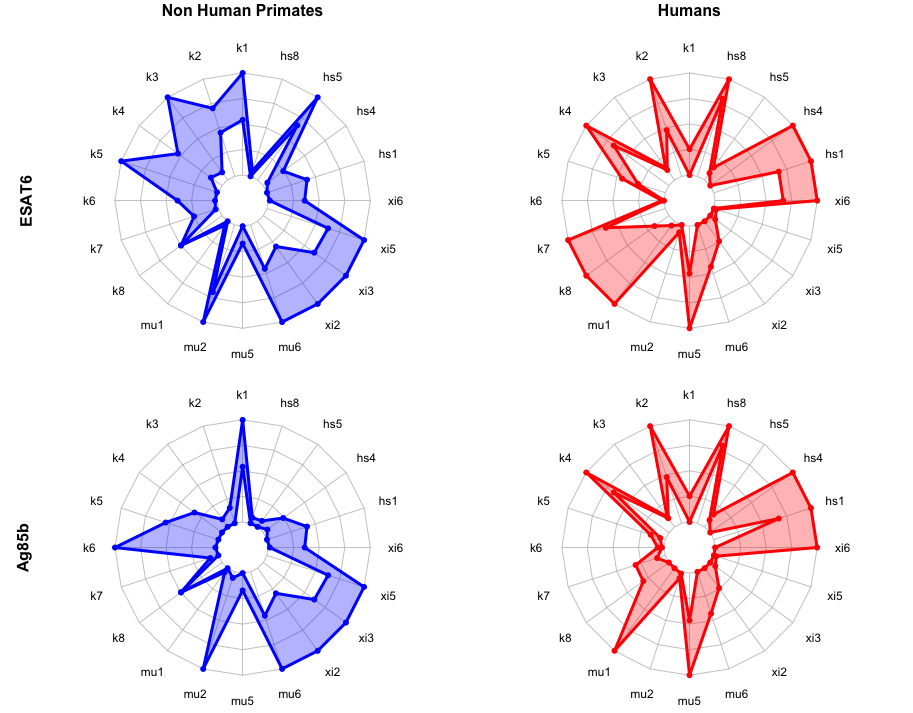


**Supplemental Figure 1:** **Radar charts reveal parameter space differences between species.** Each parameter space is represented by a blue (NHP) or red (human) band, which represents the min and max parameter value for each model fit. Each chart displays parameter names around its outside boundary, at each axis. Parameter names are ordered alphabetically starting with ‘hs1’ and ‘ending with xi6’. Points near the center of each axis represent a lower value whereas points near the outer edges of each axis represent larger values. To compare parameter ranges across species, we calculated the minimum and maximum for each axis on the charts as the minimum and maximum value for each parameter across all species and antigen specific fits.

| Parameter Name | Description |
| --- | --- |
| hs1 | Naïve CD4+ T cell recruitment half saturation |
| hs4 | Precursor CD4+ T cell proliferation half saturation |
| hs5 | Precursor CD4+ T cell differentiation half saturation |
| hs8 | Central Memory CD4+ T cell recruitment half saturation |
| k1 | Naïve CD4+ T cell recruitment rate |
| k2 | Naïve CD4+ T cell priming rate |
| k3 | Central Memory CD4+ T cell reactivation rate |
| k4 | Precursor CD4+ T cell proliferation rate |
| k5 | Precursor CD4+ T cell differentiation to effector T cell |
| k6 | Precursor CD4+ T cell differentiation to central memory T cell |
| k7 | Effector CD4+ T cell differentiation to effector Memory |
| k8 | Central Memory CD4+ T cell recruitment rate |
| mu1 | Effector CD4+ T cell death rate |
| mu2 | Effector memory CD4+ T cell death rate |
| mu5 | APC death rate |
| mu6 | Precursor CD4+ T cell death rate |
| xi2 | Naïve CD4+ Lymph Efflux |
| xi3 | Effector CD4+ Lymph Efflux |
| xi5 | Central Memory CD4+ Lymph Efflux |
| xi6 | Effector memory CD4+ Lymph Efflux |

**Supplementary Table 2: Parameter names in radar charts.** The leftmost column shows the name of each parameter. The rightmost column displays a short description of each parameter.
