# Supplementary material for: Integrating Non-human Primate, Human, and Mathematical Studies to Determine the Influence of BCG Timing on H56 Vaccine Outcomes
Source: Front Microbiol. 2018 Aug 17;9:1734. doi: 10.3389/fmicb.2018.01734 (PMC6109686; doi:10.3389/fmicb.2018.01734)
Supplement: Supplementary file 3 [file Data_Sheet_1.DOCX]

Supplementary Material 1

Mathematical model describing cell population dynamics in two compartments – the blood and lymph node – of the model described in Figure 2

Louis Joslyn, Elsje Pienaar, Robert M. DiFazio, Sara Suliman, Benjamin M. Kagina, JoAnne L. Flynn, Thomas J. Scriba, Jennifer J. Linderman^*^, Denise E. Kirschner^*^

*** Correspondence:**

Denise E. Kirschner: kirschne@umich.edu , Jennifer J. Linderman: linderma@umich.edu

# Introduction

This supplementary text describes the system of ordinary differential equations (ODEs) for CD4+ antigen-specific T cells dynamics.

Measure units are cell count in the lymph compartment and cell/mm^3^ in the blood compartment. However, upon comparison to experimental data, we converted antigen-specific T cell units into percentages of total (specific and nonspecific) CD4+ T cells in the blood. The term α , represents the volume of blood in μL and is used for scaling cells when they traffic between the blood compartment and the lymph compartment. All parameters are defined with units, values, and references provided in Supplementary Table 1.

# Lymph Node

Antigen presentation and priming in lymph node compartment is driven by the following equation

$\frac{dAPC}{dt}= -\mu_{5}APC$

which tracks antigen presenting cells (APCs) in the lymph node at any time during or after vaccination. If the number of APCs doesn’t increase (a vaccination event would be an example of increasing the APC population), the APC number decreases following an exponential decay, at the rate μ_5_.

Naïve T cells (Eqn. ) represented by ($N_{4}^{LN}$) are recruited to the lymph node at a rate (*k_1_*) dependent on cytokine production in the lymph node. Since we do not track cytokines in the lymph node model, we use APC also as a proxy for cytokine production (modeled as a Michaelis-Menten term in Eqn. ). Other terms included influx ($\xi_{1}$) and efflux ($\xi_{2}$), as well as mass action priming to precursor cells (*k_2_*).

$\frac{dN_{4}^{LN}}{dt}= \alpha\left( V_{primeN}+V_{Ninflux} \right)- V_{Nefflux}-V_{NdiffP}$

$$V_{primeN}= k_{1}N_{4}^{B}\left( \frac{APC}{APC+{hs}_{1}} \right)$$

$$V_{NdiffP}= k_{2}N_{4}^{LN}APC$$

$$V_{Nefflux}= \xi_{2}N_{4}^{LN}$$

$$V_{Ninflux}= \xi_{1}N_{4}^{B}$$

Precursor CD4+ T cells ($P_{4}^{LN}$) (Eqn. ) are generated through priming of antigen-specific naïve T cells (*k_2_*) as well as through re-activation of antigen-specific central memory T cells (*k_3_*); both processes are expressed as mass action terms. Proliferation is modeled as logistic growth.

$\frac{dP_{4}^{LN}}{dt}=\left( V_{NdiffP}+ V_{CMdiffP} \right)+ V_{prolif}-V_{PdiffE}-V_{PdiffCM}-\mu_{6}P_{4}^{LN}$

$$V_{CMdiffP}= k_{3}{CM}_{4}^{LN}APC$$

$$V_{prolif}= k_{4}P_{4}^{LN}\left( 1-\left( \frac{P_{4}^{LN}}{\rho_{1}} \right) \right)\left( \frac{APC}{APC+{hs}_{4}} \right)$$

$$V_{PdiffE}= k_{5}P_{4}^{LN}\left( \frac{APC}{APC+{hs}_{5}} \right)$$

$$V_{PdiffCM}= k_{6}P_{4}^{LN}\left( 1-\left( \frac{APC}{APC+{hs}_{5}} \right) \right)$$

A Michaelis-Menten term based on antigen stimulation (APC levels) was used to adjust proliferation (k_4_) and differentiation rates (k_5_ and k_6_). The likelihood of precursor cells differentiating into effector cells is directly proportional to the amount of antigen stimulation (k_5_). The opposite assumption was applied to the likelihood of precursor cells differentiating into central memory (k_6_). A death term (μ_6_) ensured that the precursor population did not persist in the absence of infection. No precursor populations exit the lymph node.

Effector CD4+ T cells are modeled in Eqn. , as $E_{4}^{LN}$:

$\frac{dE_{4}^{LN}}{dt}= V_{PdiffE}-V_{Eefflux}- V_{EdiffEM}$

$$V_{Eefflux}= \xi_{3}E_{4}^{LN}$$

$$V_{EdiffEM}= k_{7}E_{4}^{LN}$$

Terms in the equation include efflux to the blood ($\xi_{3}$), and a linear differentiation to the effector memory T cell population (*k_7_*). We assumed that no effector T cells die in the lymph node (they can die in the blood).

Similar to naïve cells, central memory T cells (Eqn. ) are recruited to the lymph node (k_8_) in addition to an influx rate ($\xi_{4}$). Other terms include differentiation from precursor cells (k_6_), reactivation to precursor cells (k_3_) and efflux into the blood (ξ­­­_5_). Given their relatively long lifespan compared to the length of the *in-silico* simulation (i.e., 200 days at most) we do not have a death term in Eqn. , as ${CM}_{4}^{LN}$:

$\frac{d{CM}_{4}^{LN}}{dt}= \alpha\left( V_{primeCM} + {}_{4}{CM}_{4}^{B} \right)+V_{PdiffCM}- V_{CMdiffP}-V_{CMefflux}$

$$V_{primeCM}= k_{8}{CM}_{4}^{B}\left( \frac{APC}{APC+ {hs}_{8}} \right)$$

$$V_{CMinflux}= {}_{4}{CM}_{4}^{B}$$

$$V_{CMefflux}= {}_{5}{CM}_{4}^{LN}$$

Effector memory cell formation is described in Eqn. , as ${EM}_{4}^{LN}$. A linear term captures the differentiation of CD4+ effector T cells into CD4+ effector memory (k_7_). The last term represented efflux to the blood ($\xi_{6}$). Due to the longevity of these cells, we did not introduce a death term in the lymph node. Like effector T cells, effector memory T cells do not enter the lymph node directly from the blood.

$\frac{d{EM}_{4}^{LN}}{dt}= V_{EdiffEM}-V_{EMefflux}$

$$V_{EMefflux}= {}_{6}{EM}_{4}^{LN}$$

# Blood

For the blood compartment, we track 4 different T cell antigen-specific phenotypes. The antigen-specific naïve CD4+ T cell blood population is modeled by Eqn. ($N_{4}^{B}$). We have terms for a constant source supplied from the thymus (multiplied by the antigen-specific frequency λ, i.e. $\lambda s_{N_{4}}$) to track specific and non-specific cells, migration from the lymph node ($\xi_{2}$), extra recruitment to the lymph node (k_1_), migration to the lymph node ($\xi_{1}$), and death (μ_8_).

$\frac{dN_{4}^{B}}{dt}= \lambda s_{N_{4}}+ \alpha^{-1}V_{Nefflux} - V_{primeN}- V_{Ninflux} - \mu_{8}N_{4}^{B}$

The values for $s_{N_{4}}$ and μ_8_ are chosen to maintain equilibrium in the total Naïve T cell populations (based on the initial conditions taken from the NHP blood data in previous work [1]).

Eqn. describes effector CD4+ T cells dynamics ($E_{4}^{B}$) in the blood with two terms: migration from the lymph node ($\xi_{3}$) and death (μ_1_).

$\frac{dE_{4}^{B}}{dt}= \alpha^{-1}V_{Eefflux}- \mu_{1}E_{4}^{B}$

Central memory cells in the blood (Eqn. ) (${CM}_{4}^{B}$) migrate from ($\xi_{5}$) and to the lymph node ($\xi_{4}$). Central memory cells are not recruited to the site of infection.

$\frac{d{CM}_{4}^{B}}{dt}= \alpha^{-1}V_{CMefflux}- V_{CMinflux}- V_{primeCM}$

Effector memory cells in the blood (Eqn.) (${EM}_{4}^{B}$) are modeled by two terms: migration from the lymph node ($\xi_{6}$) and death (μ_2_). Similar to effector cells these were recruited to the site of infection.

$\frac{d{EM}_{4}^{B}}{dt} = \alpha^{-1}V_{EMefflux}- \mu_{2}{EM}_{4}^{B}$

# Non-antigen-specific CD4+ lymphocytes

Our computational model similarly keeps track of non-specific T cells. However, non-antigen-specific T cells do not respond to antigen, therefore, no priming occurs in any cell population and no precursor cells are generated. Also, since we assume neither effector nor effector memory T cells enter the lymph compartment from the blood, we do not model effector or effector memory cell populations within the lymph node compartment (as shown in Figure 2). The production of the non-specific effector cells was modeled as a source term in the blood compartment and was included to meet the assumption that the previous pre-infection data describes homeostasis. The equations for non-antigen-specific CD4+ T cells are shown below. Moreover, including non-antigen-specific cells in the model makes model predictions more realistic due to the total cell numbers more accurately reflecting the actual numbers in blood.

*Naïve CD4+ non-antigen-specific* ($N_{nc4}^{LN}$)

$\frac{dN_{nc4}^{LN}}{dt}=\alpha\left(k_{1}N_{nc4}^{B}\left(\frac{APC}{APC+hs_{1}}\right)+\xi_{1}N_{nc4}^{B}\right)-\xi_{2}N_{nc4}^{LN}$ (1.11)

*Central Memory CD4+ non-antigen-specific - LN* (${CM}_{nc4}^{LN}$)

$\frac{dCM_{nc4}^{LN}}{dt}=\alpha\left(k_{8}CM_{nc4}^{B}\left(\frac{APC}{APC+hs_{8}}\right)+\xi_{4}CM_{nc4}^{B}\right)-\xi_{5}CM_{nc4}^{LN}$ (1.12)

*Naïve CD4+ non-antigen-specific – Blood* ($N_{nc4}^{B}$)

$\frac{dN_{nc4}^{B}}{dt}=(1-\lambda)s_{N_{4}}+\alpha^{-1}\xi_{2}N_{nc4}^{LN}-k_{1}N_{nc4}^{B}\left(\frac{APC}{APC+hs_{1}}\right)-\xi_{1}N_{nc4}^{B}-\mu_{8}N_{nc4}^{B}$ (1.13)

*Effector CD4+ non-antigen-specific - Blood* ($E_{nc4}^{B}$)

$\frac{dE_{nc4}^{B}}{dt}=s_{E_{nc4}}-\mu_{1}E_{nc4}^{B}$ (1.14)

As non Mtb-specific effector cells in the blood must be produced somewhere in the body, they are modeled as source and a death rate equal to that of their antigen-specific counterparts

*Central Memory CD4+ non Mtb-specific – Blood* (${CM}_{nc4}^{B}$)

$\frac{dCM_{nc4}^{B}}{dt}=\alpha^{-1}\xi_{5}CM_{nc4}^{LN}-\xi_{4}CM_{nc4}^{B}-k_{8}CM_{nc4}^{B}\left(\frac{APC}{APC+hs_{8}}\right)$ (1.15)

*Effector Memory CD4+ non-antigen-specific - Blood* (${EM}_{nc4}^{B}$)

$\frac{dEM_{nc4}^{B}}{dt}=s_{EM_{nc4}}-\mu_{2}EM_{nc4}^{B}$ (1.16)

# Works Cited

[1] S. Marino and D. Kirschner, “A Multi-Compartment Hybrid Computational Model Predicts Key Roles for Dendritic Cells in Tuberculosis Infection,” *Computation*, vol. 4, no. 4, p. 39, 2016.
